# PFAS in Nigeria: Identifying data gaps that hinder assessments of ecotoxicological and human health impacts

**DOI:** 10.1016/j.heliyon.2024.e29922

**Published:** 2024-04-21

**Authors:** Kenneth Nonso Kikanme, Nicole M. Dennis, Ochuko Felix Orikpete, Daniel Raphael Ejike Ewim

**Affiliations:** a Department of Environmental Toxicology, Texas Tech University, USA; b Department of Environmental Sciences, University of California, Riverside, USA; c Centre for Occupational Health, Safety and Environment (COHSE), University of Port Harcourt, Choba, Rivers State, Nigeria; d Department of Mechanical Engineering, Durban University of Technology, South Africa

**Keywords:** Per- and polyfluoroalkyl substances (PFAS), Ecotoxicological hazards, Neurological impacts, Environmental policy, Sustainable practices

## Abstract

This review examines the extensive use and environmental consequences of Per- and Polyfluoroalkyl Substances (PFAS) on a global scale, specifically emphasizing their potential impact in Nigeria. Recognized for their resistance to water and oil, PFAS are under increased scrutiny for their persistent nature and possible ecotoxicological risks. Here, we consolidate existing knowledge on the ecological and human health effects of PFAS in Nigeria, focusing on their neurological effects and the risks they pose to immune system health. We seek to balance the advantages of PFAS with their potential ecological and health hazards, thereby enhancing understanding of PFAS management in Nigeria and advocating for more effective policy interventions and the creation of safer alternatives. The review concludes with several recommendations: strengthening regulatory frameworks, intensifying research into the ecological and health impacts of PFAS, developing new methodologies and longitudinal studies, fostering collaborative efforts for PFAS management, and promoting public awareness and education to support sustainable environmental practices and healthier communities in Nigeria.

## 1 Introduction

Human activities have long affected both rural and urban ecosystems, causing ongoing apprehension for environmental scientists. As new and potentially hazardous pollutants are detected in environmental samples, laws protecting the environment must be revised accordingly [1]. Per- and polyfluoroalkyl substances (PFAS) are a group of chemicals characterized by their highly fluorinated aliphatic nature, which involves a chain of hydrocarbons wherein the hydrogen atoms are replaced with fluorine atoms. Perfluoroalkyls are fully substituted with fluorine atoms and are denoted as C_n_F_2n+1_, where ‘n' represents the number of carbon atoms. This unique chemical makeup, as described by Buck et al. [2], confers upon PFAS their distinctive properties, such as resistance to water, stains, and heat [3,4], high stability, and resistance to degradation, both chemically and biologically. Due to their unique properties, PFAS have integrated themselves into the fabric of modern industry and consumer goods with numerous applications, from the production of non-stick cookware and water-repellent fabrics to use in firefighting foams and a variety of other products that require durability against harsh conditions [5,6]. Since Nigeria is heavily engaged in processing and recycling electronic waste, it is possible that the contamination and exposure risk of PFAS could be increased in the region [7].

In 1980, it was discovered that the blood of 3 M employees contained ten times more organic fluorine compared to the rest of the population [8]. Currently, data from the National Health and Nutrition Examination Survey revealed that exposure to PFAS is widespread, with about 97 % of Americans having at least one type of PFAS detectable in their blood resulting from environmental vs occupational exposure [9,10]. A study in Korea confirmed the presence of PFAS in human blood [11] as did a study conducted in Germany over the period from 2009 to 2019, showing a spectrum of 37 PFAS in 100 human plasma samples [12]. Many PFAS, especially perfluoroalkyl acids (PFAAs), have been found worldwide [13] such as in human urine in the USA [14] and in human hair in Spain and Belgium [15]. The mean concentrations of perfluorooctanoic acid (PFOA) and perfluorooctane sulfonate (PFOS) in breast milk, whether estimated or measured, exceeded recommended screening values for children, often surpassing them by more than two orders of magnitude in Canada and the USA [16]. In developing countries, there is a dearth of similar exposure information necessary for proper risk assessment and regulation; however, one study in South Africa confirmed the presence of PFAS in breast milk of nursing mothers [17], highlighting the need for additional investigation in under-studied regions.

The ubiquitous presence of PFAS in the air, drinking water, and food represents a significant hazard in diverse settings from rural to suburban and urban regions and has been shown to disproportionately affect underprivileged communities [5,18]. This is of particular concern to specialists in developing countries such as Nigeria, partially because rainwater is often used as an alternative to potable water in many communities for domestic purposes [19]. The presence of PFAS in the blood of most humans tested, prompted concerns regarding their occurrence, fate, transport, and toxicological risk, which produced a plethora of PFAS literature that raised alarms about the safety of continued PFAS production and use. However, the bulk of the available PFAS literature is exclusive of data from developing countries.

The rationale for this review lies in the urgent need to consolidate the expansive knowledge available regarding PFAS and to reconcile their industrial benefits with the ecological and health risks they pose in order to encourage additional PFAS research and protective regulation in developing countries, such as Nigeria. While extensive studies have been conducted in more industrialized nations, a significant knowledge gap remains regarding the presence and effect of PFAS in developing regions worldwide [20]. Therefore, understanding the extent of PFAS use, environmental contamination, and potential human health impacts in under-studied regions like Nigeria is necessary. This shortage of region-specific PFAS research hinders the ability to assess risk and implement appropriate mitigation strategies, making it essential to investigate PFAS in these regions. Furthermore, in developing countries such as Nigeria, there is generally a low level of awareness and inadequate technology for monitoring and detecting emerging pollutants such as PFAS. Therefore, the objectives of this review are twofold: firstly, to compile and synthesize the current state of knowledge about the environmental distribution, persistence, and toxicological profile of PFAS; and secondly, to evaluate how this knowledge informs the regulatory landscape, public health policy, and the direction of future research in Nigeria. By achieving these aims, this paper seeks to provide a definitive resource for stakeholders across sectors, from environmental scientists and policymakers to industrial leaders and public health officials, and to facilitate informed decision-making and strategic planning in the face of the challenges posed by PFAS in developing countries.

## 2 Methods

This review was conducted with stringent criteria for inclusion, focusing on peer-reviewed articles that provide empirical data or comprehensive reviews on the environmental presence, toxicity, human health implications, and policy responses to PFAS, with more emphasis on Nigeria. The literature search utilized various scientific databases, including PubMed, Web of Science, Scopus, and Google Scholar, to ensure a comprehensive collection of relevant studies. Key search terms used in combination were “PFAS,” “perfluoroalkyl,” “polyfluoroalkyl,” “perfluorinated compounds,” “fate,” “occurrence,” “transport,” “ecotoxicity,” “environmental health,” “human exposure,” “regulation,” and “risk assessment.” The time frame for the search was set from the year 1990 to the present, to capture the most recent advances and discussions in the field, while also acknowledging the historical context of PFAS development and use.

The data synthesis approach involved a narrative review, summarizing, and discussing the findings from the selected studies. This was structured around major themes such as chemical properties of PFAS, routes of environmental entry, detection methods, effects on human health and wildlife, and existing regulatory measures. The synthesis aimed to draw connections between the various aspects of PFAS research, highlighting trends, consensus, and disparities in the current understanding to provide clear and actionable insights. The review was also critical, examining the methodologies of studies to assess the robustness and reliability of their conclusions. All efforts were made to ensure that the synthesis was balanced, comprehensive, and reflective of the breadth of research on PFAS.

## 3 PFAS classification, chemical properties, and uses

### 3.1 Select PFAS family compounds and their chemical properties

**Fig. 1:**
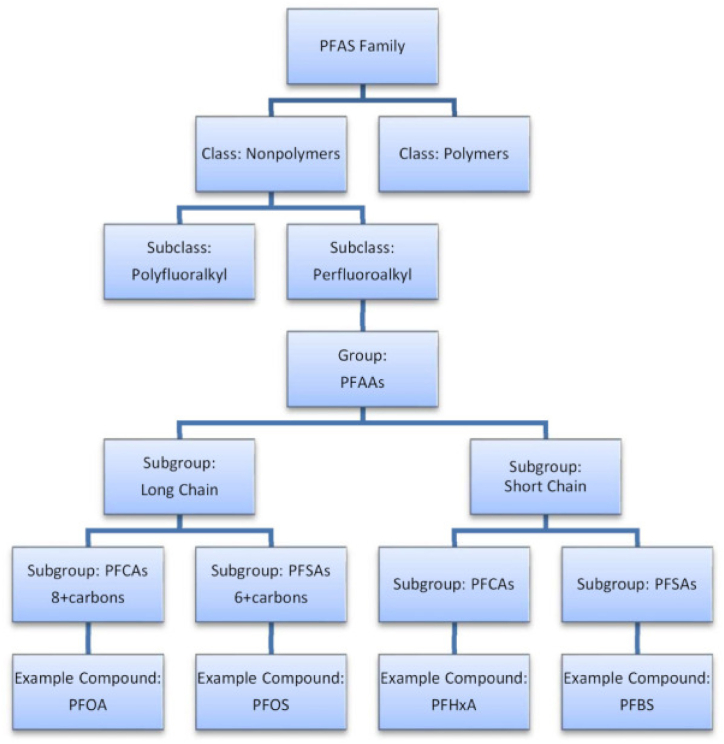
Taxonomic hierarchy of PFAS family compounds [28].

Per- and polyfluoroalkyl substances are a large family of synthetic compounds characterized by a chain of carbon atoms fully or partially fluorinated, conferring them with their unique chemical stability and resistance to degradation [21]. The carbon-fluorine bond, one of the strongest in organic chemistry, endows PFAS with hydrophobic and lipophobic properties, making them widely spread but unevenly distributed in the environment [22]. These compounds also exhibit thermal stability and high surface activity, which have been exploited in various applications [23]. The common PFAS family compounds are shown in Fig. 1. The precise count of distinct PFAS remains unclear, with estimates indicating several thousand types [24]. While it was previously estimated that over 4,800 distinct PFAS were in production, a new study by Johnson et al. [25] suggests that this number is significantly higher, with more than 7,000 different PFAS identified. Correctly classifying these compounds is essential for researching their effects on health and the environment [26]. Among them, PFAAs receive the most attention and are typically divided into two categories based on their structure: long-chain PFAAs, which have seven or more carbon atoms, and short-chain PFAAs, with fewer than seven [27]. Despite their minority status within the broader PFAS group, long-chain perfluoroalkyl carboxylic acids (PFCAs) and perfluoroalkyl sulfonic acids (PFSAs) garner significant focus from international and local research and regulatory bodies, owing to their reported environmental and health consequences [28].

### 3.2 Applications and uses of PFAS

The global production and usage trends of PFAS have shown a significant increase, particularly in the last few decades, as their applications have diversified. Although certain PFAS compounds, such as PFOA and PFOS, have been phased out in many countries due to health concerns, the production of alternative PFAS compounds continues to grow. The shift towards short-chain PFAS, presumed to be less bioaccumulative and with a shorter environmental half-life, has not necessarily alleviated environmental concerns, as the toxicity and persistence of these alternatives remain under scrutiny.

Historically, PFAS were first synthesized in the 1930s and later gained prominence with the development of polytetrafluoroethylene (PTFE), better known by its brand name, Teflon, in the 1940s [29]. Teflon is used to coat frying pans and pots to reduce friction and create non-stick surfaces, allowing for cooking with minimal sticking and easier cleaning. The non-stick properties arise from the ability of PFAS molecules to repel both water and oil [30]. Since then, PFAS usage has expanded vastly, finding roles in an array of products worldwide such as those listed in Table 1. In Nigeria, the presence of PFAS is predominantly observed in electronic wastes and household products. Although comprehensive data on production and usage are limited, it is evident that PFAS are embedded in numerous everyday items that align with global utilization trends. Such products, crucial for their durability and resistance qualities, include non-stick cookware, waterproof textiles, and stain-resistant fabrics, all of which are commonly found in Nigerian markets.

**Table 1:** Various industrial applications of PFAS [4].

Application Area	Specific uses	Details and characteristics
**Firefighting**	- Aqueous Film-Forming Foams (AFFF) -Fire-resistant materials	-Used in extinguishing high-intensity fires. -Enhances material resistance to fire.
**Textiles**	-Waterproof clothing -Stain-resistant fabrics -Carpets and upholstery	-Provides water, stain, and oil resistance. -Prolongs durability and simplifies cleaning.
**Cookware**	-Non-stick coatings (e.g., Teflon)	-Prevents food from sticking, simplifies cleaning. -Common in pots, pans, and baking sheets.
**Food packaging**	-Grease-resistant packaging -Fast food containers -Microwave popcorn bags	-Prevents grease and moisture penetration. -Maintains food quality and packaging integrity.
**Personal care products**	-Cosmetics (lipstick, foundation) -Dental floss -Shampoos and conditioners	-Enhances texture, durability, and water resistance. -In dental floss, facilitates smooth gliding.
**Industrial processes**	-Chrome plating -Hydraulic fluids -Photolithography -Oil or gas recovery wells -Pesticide ingredient	-Resistant to heat, chemicals, corrosion. -Essential in various manufacturing processes.
**Paints and coatings**	-Aircraft and automotive paints -Protective coatings -Adhesives	-Durability, resistance to environmental stress. -Used in aviation, automotive, and industrial applications.
**Medical devices**	-Surgical instruments -Implantable devices -Surgical sheets -Contact lenses -Surgical drapes and gowns	-Non-stick, chemical-resistant properties. -Essential for medical instrument functionality and safety.
**Construction materials**	-Sealants -Flooring materials -Wire insulation -Roofing	-Water, chemical resistance. -Enhances durability of construction materials.
**Electronics**	-Semiconductors -Wire coatings -Circuit boards -Cell phones -Computer cables -Floppy disks	-Heat and chemical resistance. -Dielectric properties -Low flammability -Essential for reliability and performance of electronic components.
**Household products**	-Cleaning agents -Waxes -Polishes	-Water and oil repelling properties. -Enhances efficacy of cleaning and maintenance products.
**Automotive industry**	-Fuel hoses -Gaskets -Coatings	-Chemical and heat resistance. -Increases durability and performance of automotive components.
**Outdoor gear**	-Tents -Backpacks -Outdoor furniture	-Water and stain resistance. -Prolongs lifespan and usability in outdoor environments.
**Photographic applications**	-X-ray film -Photographic paper	-Enhances quality and durability of photographic materials. -Resistant to chemicals used in photography.

Since the 1970s, PFAS have been extensively used in firefighting, especially in the form of aqueous film-forming foams (AFFFs). These foams are particularly effective against fuel fires, as they can rapidly spread over the surface of flammable liquids, forming a thin film that deprives the fire of oxygen and cools the fuel. The primary application of PFAS in firefighting revolves around their ability to create a barrier between the fuel and the air, effectively suffocating the fire. This is particularly useful in cases of high-intensity fires such as those in aviation crashes, military settings, or industrial accidents, where rapid response is critical to prevent the spread of fire [31].

PFAS have also been integral in protecting cloth for various purposes due to their ability to repel many liquids and oils. For example, PFAS enhances the protective clothing worn by firefighters. These substances have been applied to firefighter gear to impart water and stain resistance, ensuring that the clothing remains effective and durable in harsh, wet, and sooty conditions typical of firefighting environments. This treatment typically involves coating or impregnating all layers of the gear with PFAS, from the outer shell to the moisture barriers and thermal liners [32]. Similarly, PFAS are used in the production of athletic clothing due to their ability to repel water and oil. These chemicals are applied to fabrics to create a protective barrier that prevents water from penetrating, while still allowing the material to breathe. This makes PFAS ideal for use in outdoor and performance wear, where water resistance and comfort are both desired [33]. PFAS are commonly used in the furniture industry as well for the same reasons. These chemicals are often applied to upholstered furniture, such as sofas and chairs, as well as to carpets and curtains, to protect against spills and stains. The treatment with PFAS helps to prevent liquids from penetrating into the fabric, making it easier to clean and extending the life of the furniture [34].

Additionally, PFAS are utilized in various personal care products due to their unique properties, such as creating a smooth texture, providing water and oil resistance, and enhancing product durability. In cosmetics like foundation, mascara, and lipstick, PFAS improve durability and wear-resistance, contributing to water-resistant or long-lasting attributes [35,36]. Per- and polyfluoroalkyl substances are also used in certain types of dental floss to enhance glide and durability [37]. Lotions and creams may contain PFAS to achieve a smoother texture and increase resistance to water and sweat [38,39]. Ensuring effectiveness even after swimming or sweating, PFAS are also found in sunscreens for their water-resistant properties.

Furthermore, PFAS are employed in the formulation of various types of paints. The unique properties of PFAS, such as their resistance to heat, chemicals, and oil, as well as their ability to provide a smooth, durable finish, make them valuable additives in paint products [40]. Per- and polyfluoroalkyl substances contribute significantly to the durability and robustness required to withstand extreme conditions in aircraft paints. They help create a protective layer resistant to harsh weather, UV radiation, and the mechanical stresses of flight. This results in a longer lifespan for the paint, reducing the need for frequent maintenance and repainting, which is essential for aircraft efficiency and safety. Similarly, in other paint applications, PFAS enhance performance by improving the ease of application, durability, and resistance to staining and corrosion. Their water-repellent properties also make them suitable for exterior paints in buildings and structures exposed to the elements [41,42].

Finally, PFAS are part of the ingredients used in fast food packaging due to their ability to resist grease, oil, and water. This makes them ideal for coating paper and cardboard containers, including wrappers, boxes, and bowls, in fast food restaurants. The PFAS coating prevents grease from fast foods like burgers, fries, and pizzas from soaking through the packaging, maintaining the integrity and cleanliness of the package, and in turn, the surface the packaging rests upon [43].

This global proliferation has led to widespread environmental distribution of PFAS. As a result, understanding the full extent of PFAS properties, application, and uses by region is crucial for assessing the long-term environmental and health risks associated with their presence and further forms the foundation for developing effective regulatory and remediation strategies. These data are required, yet lacking for under-studied regions like Nigeria, hindering the development of effective regulatory measures. Therefore, further studies are required to gain a better understanding of the regulatory measures needed in Nigeria.

## 4 Environmental distribution, persistence, and exposure routes

**Fig. 2:**
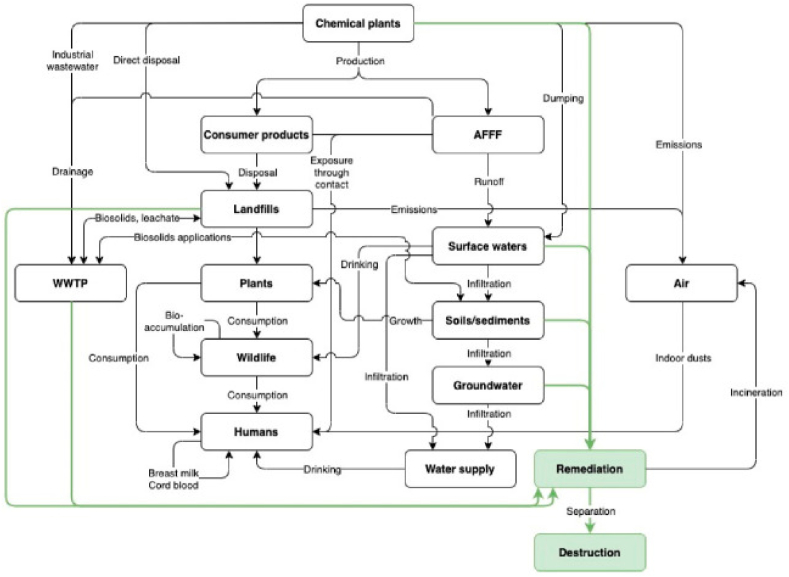
Routes of environmental discharge and exposure to PFAS [21].

The dissemination of PFAS into the environment is primarily through industrial emissions, wastewater treatment plant effluents, and the use of PFAS-containing products that gradually leach these chemicals into soil and water systems. Landfill leachates also represent a significant source, as PFAS are components of many disposed consumer goods and industrial waste. The surfactant characteristics of PFAS cause them to partition at air-water interfaces, contaminating groundwater in the process [44]. In aquatic environments, PFAS tend to bind with proteins rather than fats, which means they can accumulate in organisms differently than typical organic pollutants [45]. Once released, PFAS exhibit distinct environmental fate and transport behaviors. They can travel long distances in the environment due to their high solubility in water despite their overall chemical resistance to breakdown [46]. This mobility allows PFAS to contaminate various ecosystems, including those far from the original release point. Fig. 2 gives a detailed picture of how PFAS find their way into ecosystems and organisms.

The persistence of PFAS is due to their strong carbon-fluorine bonds, which resist photolytic, biological, and hydrolytic degradation [47]. As a result, PFAS can remain intact in the environment for years, if not decades, earning them the moniker “forever chemicals” [48]. The stability that makes PFAS valuable in industrial applications also makes their degradation in the environment exceptionally challenging [49]. Traditional environmental remediation techniques are often ineffective at breaking down PFAS, leading to the need for specialized, often costly, treatment methods. This makes bioaccumulation of PFAS a serious concern, especially for long-chain compounds that have been found to persist in the tissues of living organisms, leading to biomagnification through food webs [50]. This process results in higher concentrations of PFAS in higher trophic level species, including humans, posing significant risks to wildlife and human health. Even low-level exposure can be problematic due to the cumulative nature of these substances [51].

**Fig. 3:**
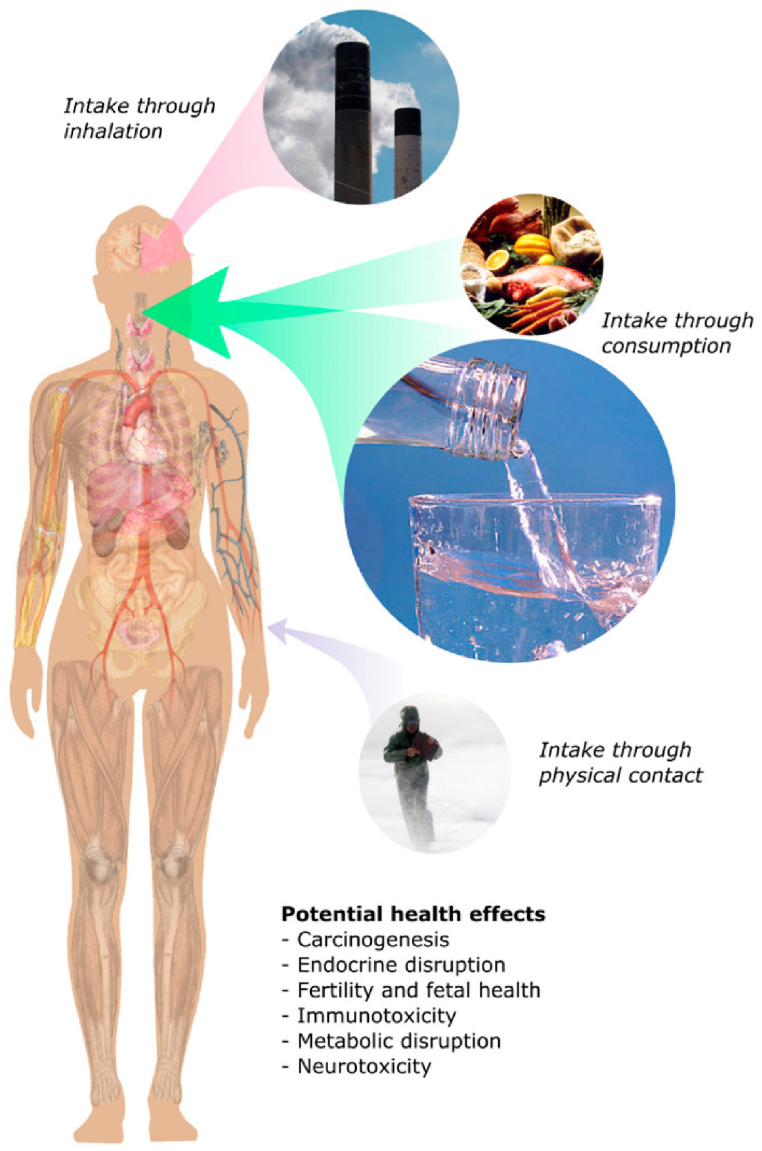
Routes of direct human exposure to per- and polyfluoroalkyl substances (PFAS) and associated health risks [21].

Direct routes of human exposure are shown in Fig. 3 and include but are not limited to the widespread use of PFAS in protective clothing, athletic clothing, non-stick cookware, food packaging, and personal care products. Widespread and direct PFAS exposure leads to concerns regarding persistence, bioaccumulation, and potential toxicity. Specifically, PFAS can leach out of treated clothing and potentially be absorbed into the skin or inhaled [52]. Additionally, when Teflon is overheated, the coatings can break down and release toxic compounds into consumed foods [53]. Similarly, PFAS can be absorbed into consumed foods from treated packaging [54,55]. Also, PFAS in personal care products carries the concern of dermal absorption and inhalation during application [56]. Once the PFAS-containing products are used directly, they are then discarded and contribute to PFAS releases into air, soil, groundwater, and surrounding environments, causing a plethora of indirect exposure routes to humans and direct exposure to wildlife. Given the potential health implications of PFAS, including its links to cancer, immune system effects, and hormone disruption, PFAS exposure has become a global issue in environmental safety and health research.

The environmental persistence, pervasive distribution, and potential toxicity of certain PFAS underscore the importance of understanding their life cycle and ecological effects globally. Consequently, continued research is essential, especially in under-studied regions such as Nigeria to develop more effective strategies for managing both local and widespread PFAS contamination and mitigating its long-term effects on ecosystems and human health.

## 5 Environmental occurrence and ecotoxicological effects of PFAS

PFAS can enter the ecosystem through various sources, such as consumer and industrial products, supply chains, manufacturing activities, and disposal methods [7,56,57]. However, studies have shown that soil does not accumulate PFOS and other PFAS as much as marine organisms do [56,58]. It is also important to note that short-chain PFAAs have a lower capacity for bioaccumulation than long-chain PFAS, with PFSAs having a higher capacity for bioaccumulation than PFCAs. According to some, long-chain PFAS such as PFOA, PFOS, and others are more hazardous than their short-chain counterparts [7,56]. Regarding specific PFAS groups, Ulhaq et al. [59] and Garg et al. [7] have discovered that PFAS-containing sulfonic groups have a greater potential to adversely affect zebrafish larvae than other PFAS groups. More ecotoxicological effects of PFAS have been extensively researched. Studies have highlighted the potential risks of PFAS to affect ecosystem structure and function in aquatic environments, emphasizing the need for further information about their ecotoxicological potential among multiple generations, species interactions, and mixture toxicity [60]. Furthermore, research has shown that PFAS can have adverse effects on amphibians, aquatic invertebrates, plankton, and microorganisms, indicating a broad impact of PFAS on aquatic ecosystems [61,62]. Additionally, PFAS has been associated with autoimmune-like effects in American alligators, further underlining the potential ecological risks these substances pose [63]. Moreover, chronic PFAS exposure under environmentally relevant conditions has been found to delay the development of Northern Leopard Frog larvae, indicating the detrimental effects of PFAS on wildlife [64].

Endocrine disruption is thought to be a key factor contributing to the wide-ranging adverse health effects observed in both wildlife and human studies [[65], [66], [67], [68]]. The ecotoxicological effects of PFAS on wildlife and ecosystems manifest in various forms, ranging from sub-lethal impacts such as altered reproductive and developmental processes to acute toxicity resulting in mortality. For example, aquatic organisms, particularly fish and amphibians, reportedly suffer from disrupted endocrine systems, reduced hatching success, and changes in growth patterns upon exposure to PFAS [69]. Research provides evidence that PFAS act as endocrine disruptors, causing harm to developing offspring in birds and disrupting the neuroendocrine system in terrestrial receptors [[70], [71], [72]]. Similar reproductive toxicities are observed in other terrestrial and aquatic receptors [56], with additional concerns about immunotoxicity and increased susceptibility to diseases [73]. The mechanisms of PFAS toxicity are complex and multifaceted. At the molecular level, PFAS can bind to proteins, disrupt normal cellular functions, and mimic or interfere with hormone activity, notably affecting thyroid regulation and reproductive hormones.

Several case studies highlight the presence of PFAS in environmental samples, globally. In biological matrices, PFAS were found in polar bear tissues, despite the region's remoteness from direct PFAS sources [74], showing that PFAS travel via oceanic and atmospheric routes. In the US, Robuck et al. [75] confirmed the presence of PFAS in various tissues and organs of juvenile seabirds. According to various research studies, there is evidence of increased concentrations of PFAS in polar bears, seabirds, fish, and other environmental receptors, posing a health risk to developing animals, globally, as well as a contamination risk to the food chain [[76], [77], [78]]. For environmental matrices, a comprehensive review was conducted on PFAS in global surface water that revealed high levels of PFOS and PFOA, exceeding US EPA advisory limits, and identified direct discharge and atmospheric deposition as key sources [79]. Rainwater from France also showed the presence of PFAS which prompted the authors to underscore the necessity for enhanced environmental surveillance and analytical methods [80]. In the U.S., PFAS contamination of surface waters and groundwater was discovered near industrial sites and military bases where PFAS-containing firefighting foams were used, leading to local fish advisories and wildlife exposures [[81], [82], [83], [84]].

In Nigeria, environmental and biological sampling data for PFAS are limited. Sindiku et al. [85] analyzed the levels of seven major perfluoroalkyl carboxylates (PFCAs) and three perfluoroalkyl sulfonates (PFSAs) in sludge from a local wastewater treatment plant. They found a notable presence of longer-chain vs shorter-chain PFAS, and measurable but generally lower levels of PFAS compared to other regions. This indicated a low usage of PFAS in Nigeria. Contrarily, Ololade et al. [86] found widespread distribution of PFOS and PFOA in water, porewater, and sediments across various Nigerian regions, with concentrations influenced by salinity and organic matter. These case studies and numerous laboratory and field studies underline the potential for PFAS to cause widespread ecological harm and generally encourage risk assessment, regulation, and remediation of PFAS-contaminated areas. Considering that Nigeria boasts a diverse array of natural ecosystems from semi-arid savanna to rainforests, freshwater swampland, and a coastal region with largest mangrove tract in Africa, there is an urgent need for further investigation into the occurrence, distribution, and long-term effects of PFAS exposure on biodiversity and ecosystem functions. This is especially crucial as environmental levels of PFAS continue to rise, and newer alternatives to long-chain PFAS are being developed without a full understanding of their ecotoxicological profiles.

## 6 Human exposure to PFAS and PFAS-associated health effects

The ubiquitous presence of PFAS in the environment has led to multiple exposure pathways for humans, including ingestion of contaminated water and food, use of PFAS-containing products, occupational exposure, and inhaling contaminated air. Research has shown that concentrations of PFAS in public drinking water supplies have exceeded the levels set forth in EPA health advisories, raising concerns about the potential health impacts on individuals [87]. The most concerning aspect of human exposure to PFAS is their bioaccumulative nature, which can lead to significant health issues over time. Van den Heuvel et al. [88] found that PFAS are predominantly present in plasma and well-perfused tissues such as the spleen, kidney, liver, brain, and testes, and they are primarily linked to cell membrane surfaces. Maestri et al. [89] conducted a human autopsy investigation and reported that the highest levels of PFOS were found in the kidney, blood, liver, and lungs, while the highest levels of PFOA were detected in the kidney, liver, and blood.

The adverse health effects associated with PFAS exposure have been extensively studied, with research linking PFAS exposure to detrimental health outcomes, including increased cholesterol, liver damage, immune suppression, and disruption of endocrine and reproductive systems [90]. Epidemiological studies provide a broader perspective on the potential health outcomes associated with PFAS exposure. These studies have observed associations between high levels of certain PFAS compounds and various health conditions, including certain cancers, thyroid disease, high cholesterol levels, and reproductive issues such as decreased fertility and pregnancy-induced hypertension. Clinical findings have also shown that PFAS can interfere with the body's natural hormones, which could explain some of the observed health effects [91,92]. Research has additionally shown that adverse health impacts of PFAS can be linked to carcinogenesis, neurotoxicity, cardiovascular diseases, developmental and reproductive disorders, and autoimmune-like effects [63,93]. Additionally, exposure to PFAS has been associated with increased risk of type 2 diabetes, altered immune and thyroid function, liver disease, lipid and insulin dysregulation, kidney disease, and adverse reproductive and developmental outcomes, and cancer, as highlighted by Shaikh et al. [94] and Abunada et al. [95]. Additionally, PFAS exposure has been associated with endocrine disruption, developmental health effects, and metabolic changes, as indicated by Ward‐Caviness et al. [96] and Blake et al. [97]. Sunderland et al. [98] reviewed the epidemiologic evidence for PFAS implications on cancer, immune function, metabolic outcomes, and neurodevelopment, emphasizing the broad spectrum of potential health effects associated with PFAS exposure. Previously believed to be harmless, it has become evident that long-chain PFAS accumulate in biological systems and are associated with reproductive and developmental issues, weakened immune function, and the development of tumours [99]. Moreover, associations between PFAS occurrence and multimorbidity have been observed, emphasizing the need for a comprehensive understanding of the public health impacts of PFAS [96]. There are no comparable human toxicology studies published to date conducted in Nigeria elucidating PFAS health effects.

Thyroid-disruptive effects were confirmed by Coperchini et al. [65] suggesting that PFAS exposure during pregnancy could potentially affect fetal and offspring development. Research conducted by Shrestha et al. [100] revealed that elevated levels of PFOA and PFOS in serum disrupted thyroid hormone function, making them particularly toxic compared to other chemicals. Meanwhile, Kim et al. [101] and Garg et al. [7] also found a connection between early-life exposure to PFAS and changes in thyroid function in children, resulting in lower TSH and higher FT4 or T3 levels.

PFAS-induced reproductive defects have been documented. Tarapore and Ouyang [102] also emphasized the potential hazard that PFAS, such as PFOA and PFOS, poses in increasing the likelihood of male infertility. In addition, PFAS exposure has been associated with delayed bone development, accelerated male puberty, and reduced birth weight in children, which suggests the potential impact on skeletal and reproductive health, as proposed by Guillete et al. [63] and Bartels et al. [103]. Studies by Vélez et al. [104] and Garg et al. [7] revealed that PFOA and PFHxS caused decreased reproductive success among women in Canada, and there was a positive correlation between progesterone and PFOS, PFOA in cord sera in China [7,105]. Other studies have linked PFAS in maternal serum, especially PFOS and perfluorohexane sulfonate (PFHxS), to low birth weight and early childhood adiposity [7,106]. Furthermore, exposure to high levels of PFOA and PFOS during early or mid-pregnancy can increase the risk of congenital cerebral palsy and other adverse birth outcomes in boys [7,107].

Neurological effects associated with PFAS exposure are increasingly becoming a point of concern. Several studies have suggested potential links between PFAS levels and decreased cognitive function, increased risk of neurodevelopmental disorders in children, and potential neurodegenerative effects in adults. Children born to mothers with high PFAS burdens during pregnancy experienced neuropsychological disorders and dysfunction in gross motor skills [7,108]. Starnes et al. [68] reviewed the neurological risks of PFAS, noting how PFAS accumulates in the brain and affected neurochemical and behavioral functions, particularly during developmental phases in vulnerable groups. The review emphasizes the discrepancy between high-dose experimental studies and lower chronic environmental exposures, suggesting the need for further research on the neurodevelopmental effects of realistic PFAS mixtures and concentrations found in the environment. The study by Brown-Leung and Cannon [109] examined the neurotoxic effects of PFAS, highlighting their ability to damage the blood-brain barrier, accumulate in the fetal brain, and alter critical neurotransmitter systems, which may contribute to neurological diseases and mental health disorders, posing a significant public health challenge due to their persistence in the environment and organisms. While research in this area is still emerging, the potential for PFAS to disrupt neurological health warrants further investigation, given the critical importance of neurological development and function across the human lifespan.

The immune system is another critical area affected by PFAS exposure [13]. Research indicates that certain PFAS compounds can diminish antibody responses to vaccines, increase the severity of infectious diseases, and may be linked to autoimmune diseases. The implications for immune health are particularly significant for children, as early-life exposure to immunotoxicants can have long-lasting consequences [110]. Rudzanová et al. [111] investigated the link between PFAS exposure and immune-mediated diseases (IMDs) in 309 Czech adults, finding a negative association of certain PFAS compounds with allergies and indications that sex may modify the effect of PFAS on atopic eczema. Transcriptomic analysis revealed 166 genes associated with PFAS exposure, with B cell signaling and plasma cell production identified as potential mechanisms of PFAS-induced immunomodulation, suggesting an immunosuppressive effect of PFAS on disease prevalence.

Given the wide range of adverse health effects associated with PFAS exposure, including its impact on the endocrine, reproductive, neurological, and immune systems, and the evidence of its ubiquitous presence and multiple pathways of exposure in humans, there is a critical need for comprehensive research in regions currently underrepresented in the literature, such as Nigeria. The absence of region-specific data on PFAS health effects hampers the ability to conduct accurate risk assessments and develop informed policies and interventions tailored to the unique environmental and public health contexts of these areas. Therefore, it is imperative to extend the scope of current research to include these under-studied regions, thereby enriching our understanding of PFAS impacts on human health globally and guiding the development of effective strategies to mitigate these risks.

## 7 Regulatory and public health response

Given the potential for PFAS to adversely affect human health, it is essential to continue monitoring and evaluating the risks posed by these chemicals. Public health policies and regulatory standards need to be informed by the most current and comprehensive scientific evidence available. This section of the paper reviews the current understanding of PFAS exposure pathways and associated health effects, highlighting the need for ongoing research and preventative measures to safeguard human health against the pervasive threat of PFAS.

Regulatory approaches to PFAS differ globally; some countries have completely banned specific PFAS types, whereas others have implemented usage restrictions or defined maximum permissible levels in drinking water. In the United States, the Environmental Protection Agency (EPA) has issued health advisories for specific PFAS compounds, such as PFOA and PFOS, setting non-enforceable limits for drinking water contamination [24]. States like Washington and California have taken significant steps to regulate the use of PFAS. As of July 1, 2018, Washington state banned using PFAS-containing Class B firefighting foam for training purposes, with no exemptions allowed. Furthermore, from July 1, 2020, Washington also prohibited the manufacture, sale, and distribution of such firefighting foams, although a few exemptions are in place. In addition to these measures, Washington mandated that manufacturers and sellers of PFAS-containing firefighting personal protective equipment (PPE) must inform buyers about the presence of PFAS and the reasons for their use starting from July 1, 2018 [112]. Similarly, California has been proactive in addressing PFAS concerns. The Department of Toxic Substance Control in California identified three PFAS-containing products as Priority Products under its Safer Consumer Products program. These include PFAS-containing carpets, PFAS in converted textiles and leathers (ranging from stylized garments to various home furnishing products), and PFAS in food plant fiber-based food packaging [112].

The European Union has taken a more proactive approach, classifying certain PFAS as substances of very high concern (SVHC) and imposing stringent restrictions on their use [113]. As 2023 commenced, the European Union implemented two significant legislative measures aimed at reducing human exposure to potentially hazardous levels of PFAS. The levels of PFAS in foodstuffs are now governed by Commission Regulation (EU) 2022/2388. Concurrently, the concentration of PFAS in drinking water is controlled under Directive (EU) 2020/2184, setting specific limits for these compounds [114]. Following the release of the EU's REACH (Registration, Evaluation, Authorization, and Restriction of Chemicals) proposal to restrict PFAS on February 7, 2023, it is anticipated that the EU's regulatory framework for PFAS will be implemented in 2025 and take effect in 2026 [115].

Globally, the Stockholm Convention on Persistent Organic Pollutants has facilitated international action, leading to the phase-out of PFOS and restrictions on other PFAS [116]. In 2020, Japan implemented a ban on the production and utilization of PFOS and PFOA. Similarly, New Zealand enforced a prohibition on PFAS in firefighting foams and is considering a ban on these substances in cosmetics. However, in many other countries such as Nigeria, regulations on PFAS remain non-existent or insufficiently developed [117]. In Canada, Health Canada has set guidelines for PFAS levels in drinking water, and in Australia, the government has initiated a voluntary phase-out of certain PFAS, along with imposing restrictions on their use in firefighting foam [118].

Public health advisories regarding PFAS are increasingly common as awareness of their potential health impacts grow. Health agencies have been active in assessing the risks of PFAS exposure and communicating guidelines to reduce exposure, particularly in communities close to industrial sites or areas with known contamination [119]. These advisories often include recommendations on consuming locally sourced foods, using alternative water sources, and specific guidance for populations most at risk, such as pregnant women and children. For example, the U.S. EPA has established health advisories for maximum concentrations of prominent PFAS, such as PFOS and PFOA [120]. These advisories are crucial for guiding regulatory frameworks and promoting the need for future epidemiological studies to address this environmental emergency [121].

The industry response to the PFAS issue has been varied, with some companies proactively phasing out long-chain PFAS in favor of shorter-chain alternatives believed to be less bioaccumulative and toxic [51]. However, the safety of these alternatives remains under scrutiny. Additionally, some industries have committed to voluntary phase-outs of PFAS production and are investing in developing non-fluorinated alternatives. This shift is partly due to regulatory pressure, but also to growing consumer demand for safer products. Unfortunately, preliminary studies regarding the safety of non-fluorinated PFAS alternatives show similar toxicity to wildlife as their PFAS counterparts [122,123], demonstrating the need for increased toxicity testing prior to commercial release of products containing PFAS alternatives.

Despite these efforts, the challenge of PFAS contamination remains and regulatory measures are often playing catch-up with the science. Consequently, there is a pressing need for enforceable global standards to manage PFAS production, use, and disposal. The response to ubiquitously present PFAS will require coordinated action between governments, industry, scientists, and public health organizations to protect human health and the environment from the long-term effects of these persistent chemicals. The current regulatory and public health responses to PFAS are varied globally, emphasizing the need for a more harmonized and precautionary approach to managing these substances.

## 8 Advances in detection and remediation

The presence of perfluorinated surfactants in aqueous samples and other sample matrixes was identified using a method developed by Moody et al. [124]. In 2004, Alzaga and Bayona [125] developed a highly sensitive analytical method that successfully determined PFAS levels in effluent from wastewater treatment plants. Higgins et al. [126] were able to detect some PFAS in sediment and sludge by implementing a technique that involved cleaning up sediments and sludge using solid-phase extraction, extracting analytes using solvent extraction, and injecting the extracts with internal standards into an LC/MS/MS system. Later, in 2006, Schultz et al. [127] fabricated a liquid chromatograph equipped with a reverse-phase column, simultaneous mass spectrometry, and electrospray ionization to identify fluorinated alkyl compounds in municipal wastewater influents and effluents. Subsequently, the detection and remediation of PFAS in the environment have been subjects of significant research and technological advancement [[128], [129], [130], [131]]. Parker et al. [132] studied how chain length and head group of PFAS affect their electrostatic interactions with anion-exchange (AE) sorbents, using liquid chromatography-mass spectrometry and calculating average electrostatic potential to predict these interactions, and determining the varying affinity of different PFAS types for AE resins in water treatment scenarios. Sophisticated analytical methods have been developed for PFAS detection, including liquid chromatography-tandem mass spectrometry (LC-MS/MS), which allows for the sensitive and specific identification of individual PFAS compounds even at low concentrations in various matrices [133]. Other techniques, such as high-resolution mass spectrometry (HRMS) and ion chromatography, have also been refined to improve the accuracy and efficiency of PFAS analysis [134,135].

In terms of remediation, strategies have been devised to address both water and soil contamination. For water treatment, methods such as granular activated carbon (GAC) filtration, ion exchange resins, and high-pressure membranes like reverse osmosis have been employed to remove PFAS from contaminated sources [136]. In soil, techniques such as soil washing, stabilization, and thermal treatment have been explored, though they can be complex and costly [137]. Moreover, the incineration of PFAS-contaminated materials has been used as a remediation strategy, although this approach has raised concerns about the formation of toxic byproducts [138]. As previously stated, the carbon-fluorine bond of PFAS molecules makes them resistant to conventional degradation processes, requiring more innovative and aggressive treatment methods. Therefore, current remediation research has focused on developing more affordable, sustainable, and effective strategies. For example, researchers are currently probing the use of advanced oxidation processes, enzymatic treatments, and emerging technologies like nano-remediation [129,[139], [140], [141], [142]]. Furthermore, Wen et al. [143] and Zhang et al. [144] are investigating the environmental fate of PFAS after remediation to ensure that treatment process byproducts do not pose additional ecological or health risks.

Despite advances in PFAS detection and remediation, limited analytical capabilities in developing countries, including Nigeria, continue to impede PFAS research progress [85,145,146]. One of the primary issues is the sheer diversity and number of PFAS compounds, which complicates detection and treatment processes as well as our ability to tease out drivers of PFAS toxicity. Fortunately, as we advance our understanding of PFAS chemistry and environmental behavior, it is likely that new and improved methods for detection and remediation will emerge. Ongoing PFAS detection and remediation efforts are promising avenues for future research [135]; however, there is still a need for cost-effective, scalable, and environmentally sound management solutions, especially in developing countries such as Nigeria, where limited resources are available for remediation.

## 9 Recommendations

The effective management of PFAS necessitates a collaborative, multi-stakeholder strategy that includes government agencies, environmental and health organizations, industrial sectors, non-governmental organizations, research institutions, local communities, individual citizens, legal entities, and international organizations on both global and local scales. Effective mitigation and remediation of PFAS contamination require a cooperative approach, where sharing information and active involvement from all relevant stakeholders are crucial to safeguard the environment and public health.

It is imperative to take immediate action to tackle PFAS contamination, especially in under-studied regions like Nigeria, ensuring the protection of both environmental and public health worldwide. Through this comprehensive review it is clear that the challenges caused by PFAS contamination are expansive, mostly due to their persistent nature in the environment and potential for significant bioaccumulation. Their ubiquitous presence poses risks to both human health and ecological systems, worldwide. Research findings indicate a clear association between PFAS exposure and various adverse health outcomes, including thyroid issues, immune system suppression, and developmental problems, raising serious questions about their continued use. Given these concerns, the following recommendations are proposed to address the complexities surrounding PFAS management in Nigeria and indeed, globally.

1. *Enhanced regulatory frameworks*: At present, Nigeria lacks comprehensive regulations for the management, use, and disposal of PFAS [147]. Existing policies may not sufficiently capture the nuances of PFAS contamination, especially considering the wide array of PFAS compounds and their diverse toxicities. There is a need for more inclusive policies that encompass the entire PFAS class and focus on their lifecycle management to minimize environmental release.
2. *Focused research on ecological and health implications*: Like most developing nations, Nigeria lacks significant research data to ascertain current ecological risk assessment of legacy PFAS, degradation products, and new replacements [7,20,85]. The contributing factor is limited analytical instrumentation and poor maintenance of available instruments. More laboratory, biological surveys, New Approach Methodologies, and epidemiological research are needed to establish the significant PFAS exposure screening benchmark data to ascertain definitive environmental and health risk outcomes.
3. *Development of effective remediation technologies*: Nigeria lacks cost-effective PFAS remedial technologies. However, some existing technologies show promise in PFAS remediation [148], and more research is required to assess their long-term effectiveness and potential ecological impacts. Innovative and sustainable remediation strategies must be developed and tested. For example, hybrid techniques suggested by Wanninayake et al. [149] in terms of energy efficiency and usage are highly effective compared to individual techniques.
4. *Comprehensive longitudinal studies*: As noted in several chronic exposure studies, to fully understand the chronic effects of PFAS exposure, there is a critical need for long-term, longitudinal studies such as those conducted by Newsted et al. [150] and Dennis et al. [71]. These studies should aim to remain biologically and environmentally relevant and attempt to elucidate PFAS chemistry, environmental behaviors, and the mechanisms of biological impact.
5. *Collaborative approach for PFAS management*: The implementation of stringent regulations, the encouragement of eco-friendlier production practices, and the shift towards safer substitutes are crucial steps in diminishing the use of PFASs and averting additional environmental pollution [147,151]. Therefore, addressing PFAS contamination globally and regionally will require a unified approach involving the government, environmental organizations, industry players, and the general public.
6. *Adoption of the Precautionary Principle*: Nigeria has no available approach to preventive action in the face of uncertainty and potential risk associated with PFAS products and contamination. A more aggressive stance on reducing and eventually phasing out the use of PFAS, particularly in consumer products and industrial processes, is necessary [152]. In policy-making, a precautionary approach should be adopted, as Wollin et al. [153] suggested.
7. *Public awareness and education*: Environmental sustainability, circular economy, and crude oil clean up awareness is growing in Nigeria. However, few or no organizations are championing PFAS awareness and the need for regulation. Hence, increasing public awareness about the risks associated with PFAS and promoting education on safer alternatives could significantly reduce exposure in Nigeria [154].
8. *Regulate the use of PFAS firefighting products:* The toxicity of PFAS aqueous film-forming foams employed as fire extinguishers is acknowledged to have detrimental effects on both aquatic and environmental receptors, as evidenced by research conducted by Da Silva et al. [155], Bursian et al. [156], and Jones et al. [157]. As a result, numerous countries have taken the step of prohibiting the use, importation, and exportation of these firefighting products [115]. However, in Nigeria, firefighting organizations continue to import these products to extinguish oil rig explosions. Therefore, it is essential to implement a mechanism to curtail this practice, such as regulating the importation and use of these products.

By implementing these recommendations in Nigeria and, indeed, globally, there is a potential to not only mitigate the current challenges posed by PFAS but also to safeguard public health and environmental integrity for future generations. The issue of PFAS is not only a matter of scientific and regulatory concern but also a broader societal challenge that necessitates immediate and concerted action from all stakeholders as well as the general public.

## 10 Conclusion

This in-depth review of PFAS has demonstrated their widespread environmental dispersion, durability, and potential for bioaccumulation, all of which present substantial risks to ecological systems and public health around the world. The review highlights the resilience of PFAS compounds against standard degradation processes and their links to a range of adverse health effects. Despite progress in detecting and mitigating these contaminants, significant challenges persist in their management. While much is known about PFAS occurrence, fate, transport, toxicity, and remediation in developed countries, there is still a dearth of similar PFAS data available in developing countries, such as Nigeria. Region-specific data is necessary for comprehensive risk assessments and encouragement of PFAS regulation in order to protect both human and environmental health in developing countries alike.

In particular, for Nigerian policymakers, this review demonstrates the urgency for more stringent regulations and comprehensive guidelines addressing the entire spectrum of PFAS chemicals. It's advisable that new policies should extend beyond restricting long-chain PFAS, to also evaluate their substitutes critically, thereby avoiding the replacement of one harmful compound with another. Implementing enhanced monitoring and stringent reporting standards is vital for tracking PFAS presence in both the environment and consumer products. The industrial sector in Nigeria is encouraged to increase transparency regarding PFAS usage and to commit to researching and developing non-fluorinated alternatives that are environmentally benign. They should further consider following the lead of other countries and request voluntary phase-outs of PFAS and should also require enhanced product labelling to enable consumers to make informed choices about products containing these chemicals. There is a crucial need for ongoing research to deepen our understanding of the long-term effects of PFAS, to refine removal methods, and to discover safer alternatives. Collaboration among scientists, regulatory bodies, and industry stakeholders is key to developing comprehensive solutions to the PFAS issue in Nigeria and abroad.

In summary, this review calls for collective efforts both globally and locally to develop safe, sustainable alternatives to PFAS, and demonstrated the immediate need for innovation, particularly in developing countries. This review further revealed that the health of Nigeria's ecosystems, wildlife, and human populations hinges on our capacity to find and apply effective responses to the PFAS challenge. As we accumulate more evidence in under-studied regions of the world such as Nigeria, Nigeria's commitment to a future free from the widespread risk of PFAS contamination must also grow. Recognizing the gravity of PFAS contamination and fostering collaborative efforts, Nigeria can set a course towards a cleaner, safer future, safeguarding the health of our citizens and conserving its natural heritage for future generations.

## Funding

No funding was obtained for this study.

## Data availability statement

This review article does not contain any new empirical data but is a synthesis of previously published literature on the topic of PFAS exposure and its health effects. Accordingly, all data analyzed during this study are cited and can be found in the public domain through the referenced articles. For specific data inquiries, readers are encouraged to consult the original sources as listed in the References section of this paper.

## CRediT authorship contribution statement

**Kenneth Nonso Kikanme:** Methodology, Conceptualization. **Nicole M. Dennis:** Supervision, Conceptualization. **Ochuko Felix Orikpete:** Writing – original draft, Investigation, Formal analysis. **Daniel Raphael Ejike Ewim:** Writing – review & editing, Resources.

## Declaration of competing interest

The authors declare that they have no known competing financial interests or personal relationships that could have appeared to influence the work reported in this paper.
